# Observation of Microvascular Perfusion in the Hegu (LI4) Acupoint Area after Deqi Acupuncture at Quchi (LI11) Acupoint Using Speckle Laser Blood Flow Scanning Technology

**DOI:** 10.1155/2012/604590

**Published:** 2012-10-31

**Authors:** Tao Huang, Li-Jian Yang, Wei-Bo Zhang, Shu-Yong Jia, Yu-Ying Tian, Guan-Jun Wang, Xiang Mu, Lu Wang, Gerhard Litscher

**Affiliations:** ^1^Institute of Acupuncture and Moxibustion, China Academy of Chinese Medical Science, No. 16 Nanxiaojie of Dongzhimen, Beijing 100700, China; ^2^Beijing Key Laboratory of Traditional Chinese Veterinary Medicine, Beijing University of Agriculture, Beijing 102206, China; ^3^Stronach Research Unit for Complementary and Integrative Laser Medicine, TCM Research Center Graz and Research Unit of Biomedical Engineering in Anesthesia and Intensive Care Medicine, Medical University of Graz, Auenbruggerplatz 29, 8036 Graz, Austria

## Abstract

The aim of this study was to investigate the traditional meridian theory using speckle laser blood flow scanning technology to observe microcirculation of the Hegu acupoint area after acupuncture stimulation on distant points. An observational study was conducted to observe the microvascular perfusion of Hegu (LI4) and control points after acupuncturing Quchi (LI11). Thirty healthy volunteers (mean age 31.6 ± 8.7 years) received deqi acupuncture on Quchi (LI11, right side), and simultaneously changes in microvascular perfusion of Sanjian (LI3), Hegu (LI4), Yangxi (LI5), and two control points were observed before, during, and after needling using a MOOR speckle laser. The results showed that the changes in microvascular perfusion of the observed points are not regular. After correction, the experiment showed that the blood perfusion on 3 meridian acupoints was increased while the perfusion on 2 control points was decreased following acupuncture stimulation, the changes at Hegu (LI4) being the statistically most significant ones. Deqi acupuncture can help in regulating the body's blood flow, with a certain degree of meridian specificity.

## 1. Background

In accordance with traditional chinese medicine (TCM), effective acupuncture could enhance people's health by clearing main and collateral channels, thus allowing the body's Yin and Yang to achieve a state of dynamic equilibrium. Usually, effective acupuncture is also called deqi acupuncture (arrival of Qi). Deqi/Qi arrival (with its uniquely human characteristics like Qigong and Yin-yang) is accepted by parts of the international academic community [1]. When inserting the needle to a certain depth, both the acupuncturist and the patient will feel something is changing, this means Qi arrival or deqi sensation. Comparing placebo and deqi acupuncture, it was found that after acupuncture stimulation on one point, the former case saw a universal but insignificant increase of transcutaneous CO_2_ emission, while the latter case showed a significant increase of transcutaneous CO_2_ emission specifically at acupoints located on the same meridian [2].

The physiological changes caused by acupuncture are multifaceted, they occur on different levels and are aimed at different targets. In previous studies, our group observed changes of microcirculation in Hegu (LI4), Neiguan (PC6), and Weizhong (UB40) areas after deqi acupuncture stimulation using laser Doppler perfusion imaging, and we found that there are 3 kinds of effects: on the acupoint areas, on meridians, and on the whole body [3–5]. After deqi acupuncture at some acupoints, the microvascular skin perfusion of some special distant acupoints showed specific changes with statistical difference compared to the control points, which is in keeping with the traditional Chinese medical acupuncture and meridian theory.

In order to further test the results described above, an experiment was conducted at the Institute of Acupuncture and Moxibustion (China Academy of Chinese Medical Sciences), to observe the blood flow changes on the Hegu (LI4) acupoint after acupuncturing Quchi (LI11) using speckle laser blood flow scanning technology. 

## 2. Materials and Methods

### 2.1. Selection of Study Participants and Acupuncture Methods

All participants gave informed consent. The experimental procedure was approved by the Ethics Committee of the Institute of Acupuncture and Moxibustion of China Academy of Chinese Medical Sciences. To avoid discrepancies in manipulation, all the acupuncture operations were performed by the same medical practitioner.

Thirty healthy volunteers (14 female, 16 male; mean age ± SD 31.6 ± 8.7 years) came from the Beijing University of Traditional Chinese Medicine and the China Academy of Chinese Medical Sciences.

**Figure 1:**
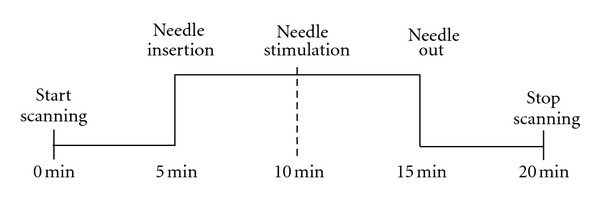
Flow diagram of acupuncture and scanning.

Acupuncture stimulation was done manually, using single-use acupuncture needles (0.30 × 40 mm, Huacheng brand, Suzhou, China). The acupoint Quchi (LI11; located in the midpoint between the lateral end of the transverse cubical crease and the lateral epicondyle of the humerus; right side), was punctured perpendicularly (needle insertion depth 15–20 mm), then needle stimulation was performed by lifting, thrusting, and rotating the needle till achieving Qi arrival. The needle was left in place for 10 min and then removed (see Figure 1).

### 2.2. Observation Methods

**Figure 2:**
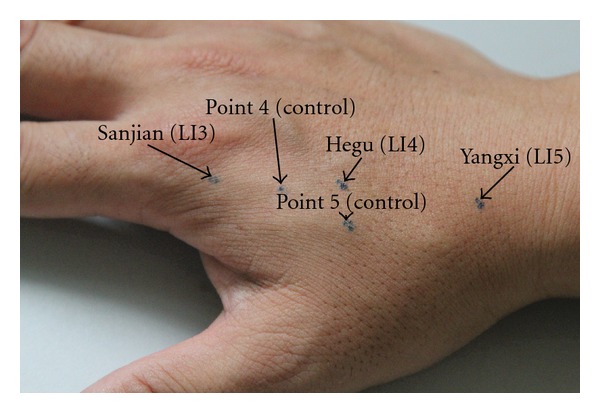
Marked points to be investigated.

The temperature of the lab was kept at 26°C, and the volunteers were asked to come into the room 5–10 mins ahead of schedule to adapt to the temperature. Then we marked point 1 (Sanjian, LI3), point 2 (Hegu, LI4), point 3 (Yangxi, LI5), point 4 (control point, located on the Large Intestine channel/meridian), and point 5 (control point, not located on the Large Intestine channel/meridian); see Figure 2.

The principle of speckle laser blood flow scanning technology is that the speckle (basically the spot of light emitted by the instrument) is formatted at the tissue surface, and the speckle will change with the moving particles (such as red blood cells). Then the information about perfusion distribution can be obtained by assessing the intensity of fluctuations. The instrument we used was a multi-point synchronization scanner Moor FLPI (Moor Instruments Ltd., Millwey, UK). Measurement parameters: scanning distance about 11 cm; sample interval 40 ms; flux time constant 0.5 s; total scanning time 20 min. For the exact procedure of the experiment, see Figure 1. During the whole experiment, the volunteers were requested to be quiet unless being asked about the needle sensation.

### 2.3. Statistical Analysis

Thirty sets of average data of the skin microvascular perfusion (Flux, in arbitrary units) were analyzed by Moor Full-field Laser Perfusion Single Point Review 2.1 (provided by Moor Instruments Ltd.), comparing the average values of the different periods (before, during, and after acupuncture).

Further, according to our past research results and to eliminate the systemic effects of acupuncture, we processed the first 4 groups of original data referenced with point 5 as a control point. The correction formula was as follows. 

During acupuncture-before acupuncture of point 1–4/during acupuncture of point 5.

After acupuncture-during acupuncture of point 1–4/after acupuncture of point 5.

Paired *t*-test was used to compare the differences between the blood flow per unit in different periods of acupuncture, with *P* < 0.05 denoted as significant.

## 3. Results

### 3.1. Analysis of the Original Data

When analyzing the average of original skin microvascular perfusion, it was noted that the blood flow of the observation points was different, with a large data standard deviation. The highest level of skin blood flow area was on Sanjian (LI3) which is situated foremost on the finger, and the lowest was on Hegu (LI4).

The blood flow of the observed points was influenced by needling Quchi (LI11), however, the changes did not reach the level of statistical significance (see Table 1). 

### 3.2. The Corrected Ratio Analysis

From the past research results we know that every kind of acupuncture stimulation—including shallow acupuncture, but also deqi acupuncture—can bring about systemic reactions and changes of microvascular perfusion. To eliminate this influence, the original data was processed using the correction method described above [6], and results are shown in Table 2. After averaging the ratio during and after acupuncture, it was found that the skin microvascular perfusion of the point 1–3 areas all increased, while that of the control points decreased. But only the change at Hegu (LI4) had statistical significance (*P* < 0.01), while the changes in other acupoints did not reach the level of statistical significance.

Comparing the ratio of microvascular perfusion of points 1–4, it showed that there was no significant difference (*P* > 0.05) among 3 channel/meridian points (Point 1–3 (LI3, LI4, LI5)). However, there was a significant difference (*P* < 0.01) between Yangxi (LI5) and control point 4 as well as between Hegu (LI4) and control point 4 (*P* = 0.0017).

## 4. Discussion

This group of experiments focuses on the role of acupuncture on microvascular function.

Microvessels provide the environment for cell growth, thus having direct impact on the function of cells and tissues. The microcirculation directly participates in the flow present in tissue (cell, blood, lymph, and tissue fluid) and is considered as a breakthrough for researching meridian phenomenon and acupuncture mechanism. In 1988, Xiu et al. reported that after acupuncturing Chize (Lu5), the skin microvascular self-discipline movement frequency of Shaoshang (Lu11) remained unchanged while the amplitude increased by more than 60%. They concluded that acupuncture could regulate microcirculation such as the microvascular vasomotion [7]. Mu et al. also proposed that the blood flow in acupoints and non-acupoints was significantly different, and acupuncture could increase the amplitude of microvascular vasomotion as well as the blood flow velocity in the acupoint area [8]. With acupuncture stimulation on Neiguan (PC6), Yuan et al. observed that the blood flow of the acupoints on the Pericardium meridian increased compared with the 4 control points on the heart and spleen meridian [9]. Sa et al. also noticed that acupuncture had a positive impact on the deep tissue of the points and control points on or on the left/right of the stomach channel/meridian [10]. 

For revealing the difference between acupoints and non-acupoints with regard to microcirculation, our group did a series of studies on skin microvascular perfusion after acupuncture stimulation using laser Doppler flowmetry, laser Doppler imaging, and laser speckle scanning technology [11, 12]. Previous studies in our laboratory showed that acupuncture could specifically enhance the related regional blood flow within a specific period of time. When needling Weizhong (UB40) and achieving Qi arrival, blood flow at the lumbar area of low back pain patients increased specifically, which is in concordance with the traditional Chinese medicinal old saying of “*Yao Bei Wei Zhong Qiu*, UB40 could solve all the problems of the lower back” [11]. When applying manual needling on Hegu (LI4) and getting Qi arrival, the volunteers' facial blood flow specifically increased, in conformity with the traditional Chinese medicinal old saying of  “*Mian Kou He Gu Shou*, LI4 could solve all the problems of the face and mouth” [12]. When acupuncturing Neiguan (PC6) with Qi arrival, the blood flow of Quze (LU5), which is located near the elbow, decreased instantly but then increased significantly [6]. This phenomenon is consistent with the results of Itaya et al. [13]. Itaya acupunctured Geshu (UB17) in rabbits, and the microvascular blood flow slightly decreased in the early stage, but when the needling stopped, the rhythmic movement of the microvessels increased quickly [13].

In previous studies at our laboratory, all experiments were conducted by stimulating acupoints in the extremities to observe the blood flow of acupoints in the proximal end on the same channel/meridian. The current experiment was conducted by stimulating the proximal end point Quchi (LI11) to observe the blood flow on the lower end of the same extremity, at Hegu (LI4), Sanjian (LI3), Yangxi (LI5), and the control points. In the current experiment and past experiments, only one point was stimulated at a time. The results showed that the increase of blood flow in 3 related meridian acupoints after needling Quchi (LI11) was statistically significant compared with that in the control points. Especially the blood flow of the Hegu (LI4) acupoint obviously changed after acupuncturing the point Quchi (LI11).

## Figures and Tables

**Table 1:** Changing of skin microvascular perfusion (in arbitrary units) of different points before, during, and after needling homolateral Quchi (LI11) in experiment 1 (*N* = 30).

Mean	Before	During	After
	acupuncture	acupuncture	acupuncture
Sanjian (LI3)	87.24 ± 55.20	91.99 ± 57.08	91.16 ± 51.75
Hegu (LI4)	57.57 ± 36.57	61.29 ± 38.55	61.28 ± 39.17
Yangxi (LI5)	63.24 ± 25.35	65.17 ± 25.32	65.48 ± 25.90
Point 4	71.20 ± 34.67	69.92 ± 32.80	68.25 ± 29.63
Point 5	65.96 ± 43.17	69.50 ± 52.69	66.03 ± 45.71

**Table 2:** The ratio of skin microvascular perfusion of Points 1–4 referenced to Point 5 before acupuncture in the experiment.

	Sanjian (LI3)	Hegu (LI4)	Yangxi (LI5)	Point 4	Point 5
During acupuncture	0.02	0.04	0.03	−0.03	0.01
After acupuncture	0.04	0.56	0.03	−0.04	−0.02
